# Synthesis and effect of substituent position, metal type on the electrochemical properties of (3-morpholin-4-ylpropoxy) groups substituted cobalt, manganese phthalocyanines

**DOI:** 10.3906/kim-2001-54

**Published:** 2020-06-01

**Authors:** Zekeriya BIYIKLIOĞLU, Hüseyin BAŞ

**Affiliations:** 1 Department of Chemistry, Karadeniz Technical University, Trabzon turkey

**Keywords:** Synthesis, phthalocyanine, cobalt, manganese, voltammetry

## Abstract

In this work, 4-(3-morpholin-4-ylpropoxy)phthalonitrile 2, 3-(3-morpholin-4-ylpropoxy)phthalonitrile 3, Co(II)Pc and Mn(III)Pcs containing (3-morpholin-4-ylpropoxy) groups at peripheral and nonperipheral positions were synthesized. Phthalonitrile derivatives (2 and 3), Co(II)Pc and Mn(III)Pcs (2a, 2b, 3a, 3b) were characterized by using FT-IR, NMR (only for 2 and 3), mass and UV–Vis (except 2 and 3) spectral data techniques. Also, electrochemistry of (3-morpholin-4-ylpropoxy) group substituted Co(II)Pc and Mn(III)Pcs were inspected by using cyclic voltammetry. Electrochemical studies show that (3-morpholin-4-ylpropoxy) group substituted Co(II)Pc and Mn(III)Pcs electropolymerized on the Pt working electrode.

## 1. Introduction

Peripheral or nonperipheral tetra-substituted phthalocyanines have been investigated in different areas owing to their chemical and thermal stability that possess physical and chemical properties [1,2]. Phthalocyanines and their derivatives have been used in many applications such as chemical and biosensor [3], solar cell, [4,5], catalyst [6], nonlinear optic [7], liquid crystal [8], catalyst [9,10], photosensitizers in photodynamic therapy (PDT) [11,12], because they have intense blue-green colour owing to the electronic delocalization of their 18-π electrons. On the other hand, electrochemical characterization of phthalocyanines is important for the electrocatalyst [13], electrosensing [14], electropolymerization [15,16], electrochromic fields [17].

Phthalocyanines bearing redox active metals (Co, Fe and Mn) have been investigated owing to their electrocatalytic properties [18]. The usage of cobalt phthalocyanines as an electrochemical sensor is of interest [19,20]. Also, Mn(III)Pcs exhibit exciting electrochemical response owing to different oxidation states of manganese ion [21]. In this work, we have synthesized Co(II)Pc and Mn(III)Pcs containing (3-morpholin-4-ylpropoxy) groups at peripheral and nonperipheral positions. It has been found that the attachment of -(3-morpholin-4-ylpropoxy) group to the phthalocyanine molecule in either a peripheral or nonperipheral position has a great effect on the electrochemical properties.

## 2. Experimental design

All information about the used equipment, materials, synthesis, electrochemistry experiments is given in the Supplementary Information.

## 3. Results and discussion

### 3.1. Synthesis and characterization

**Figure 1:**
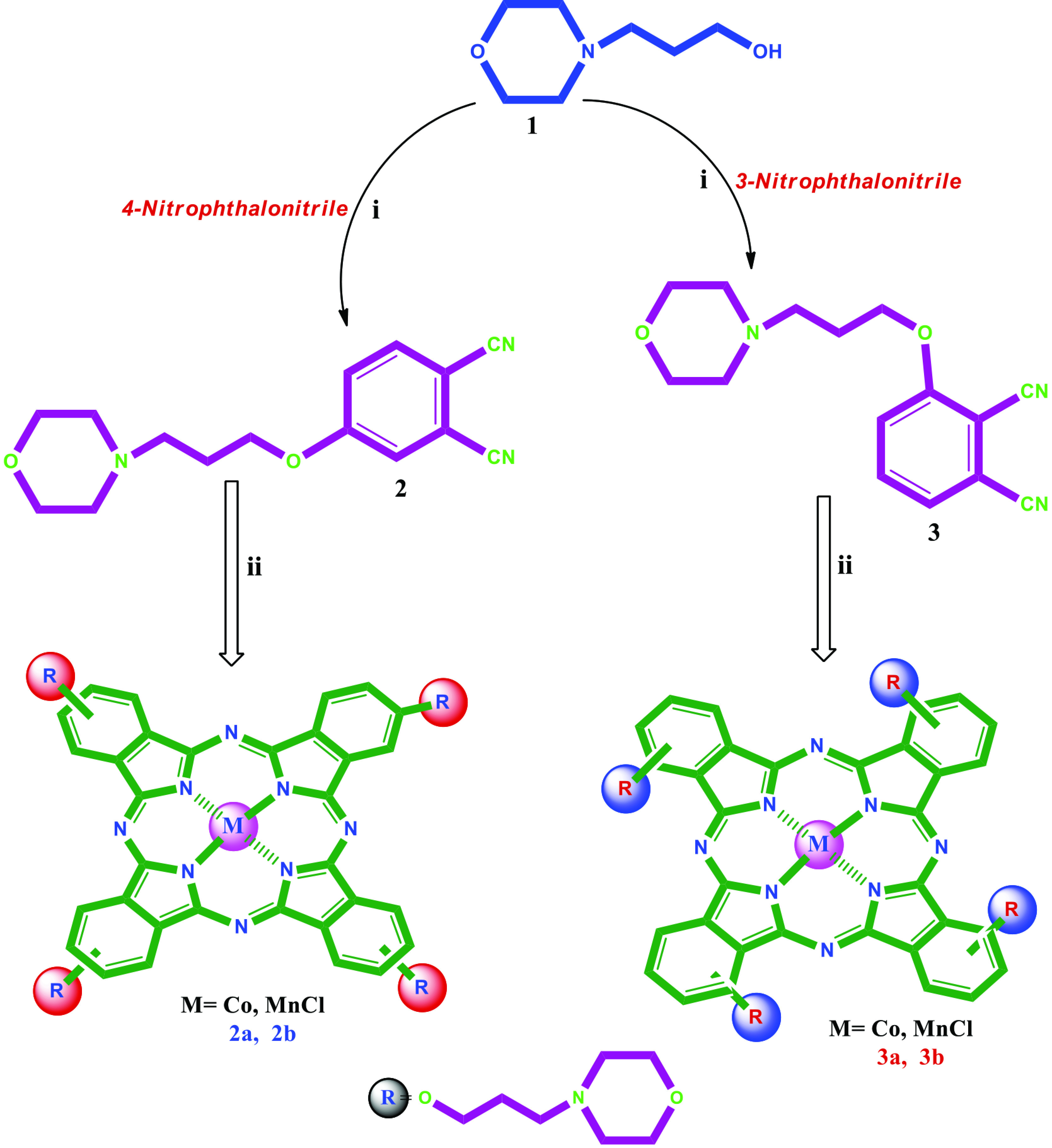
The synthesis of Co(II)Pc and Mn(III)Pcs bearing (3-morpholin-4-ylpropoxy) groups. (i) dry DMF, K_2_CO_3_, 60 °C, 96 h.

**Figure 2:**
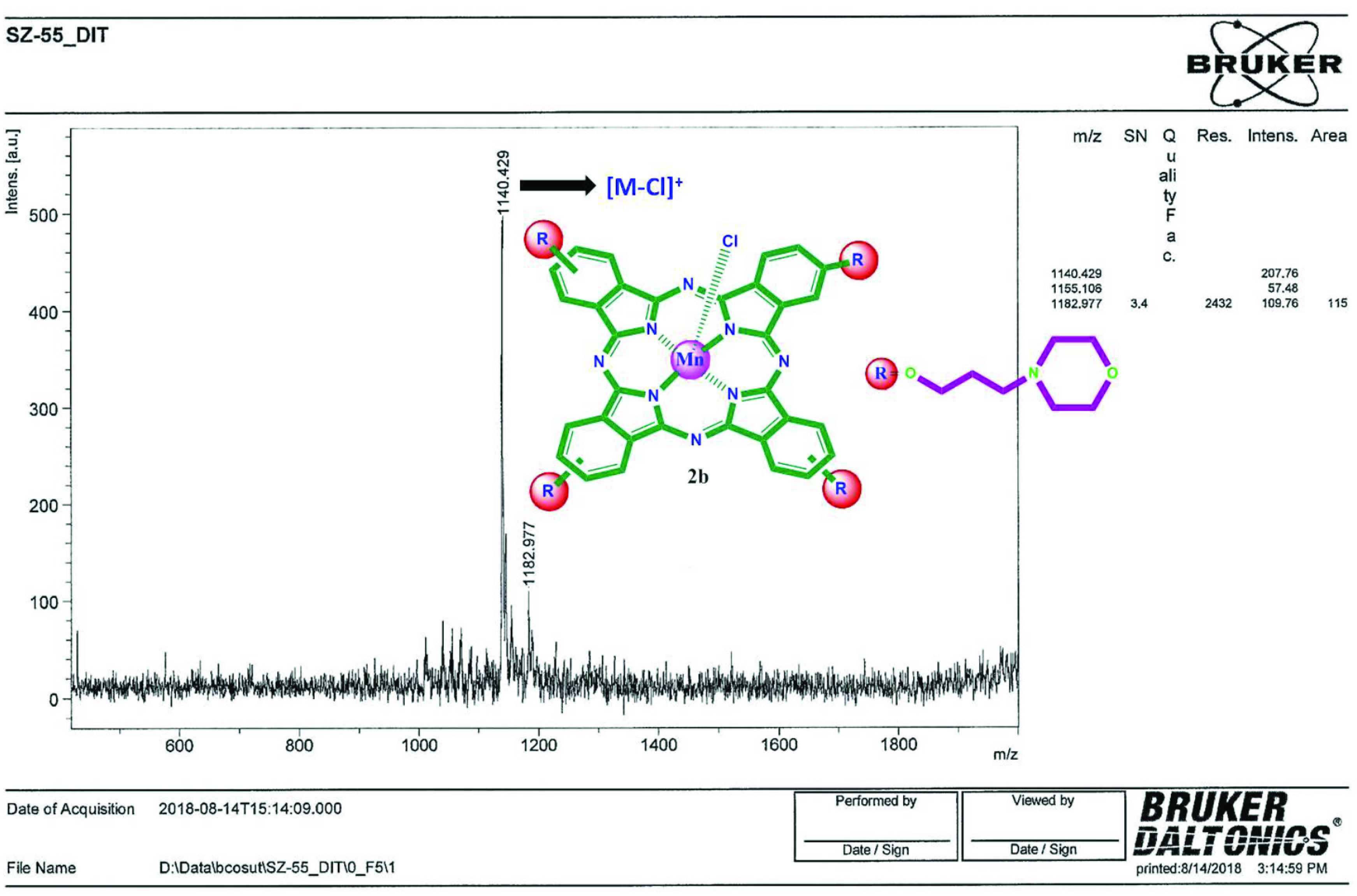
MALDI-TOF MS spectrum of 2b.

**Figure 3:**
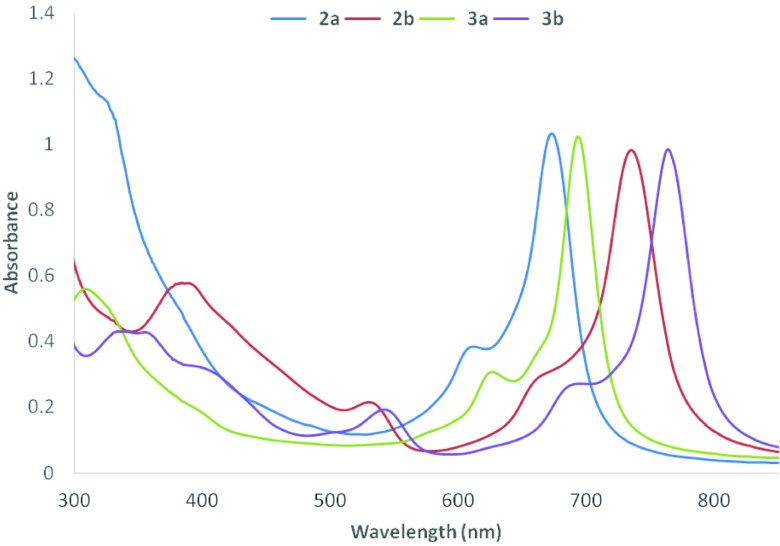
UV-Vis spectra of 2a, 2b, 3a, 3b in CHCl_3_. (Concentration: 1.00 ×10^-5^ M)

The synthesis of Co(II)Pc and Mn(III)Pcs bearing (3-morpholin-4-ylpropoxy) groups was shown in Figure 1. Firstly, 4-(3-morpholin-4-ylpropoxy)phthalonitrile (2), 3-(3-morpholin-4-ylpropoxy)phthalonitrile (3) were synthesized from 3-morpholin-4-yl-propan-1-ol in the presence of available phthalonitrile by using K2 CO3 in dry DMF [22,23]. Then, peripheral, nonperipheral tetra-(3-morpholin-4-ylpropoxy) group substituted Co(II)Pc and Mn(III)Pcs (2a, 2b, 3a, 3b) were synthesized by cyclotetramerization from 2, 3. In the IR spectrum of 2, 3, stretching vibrations of C≡N groups at 2230 (for 2), 2228 (for 3) cm^-1^ occurred at expected frequencies, respectively. In ^1^H-NMR spectrum of 2 and 3 in CDCl3 , aromatic protons appeared at 7.72–7.20 and 7.66–7.30 ppm. In the ^13^C-NMR spectrum of 2 and 3, nitrile carbon atoms were resonated at δ 115.75, 115.32 ppm (for 2) and 116.98, 113.00 ppm (for 3). The molecular ion peak of 4-(3-morpholin-4-ylpropoxy)phthalonitrile (2), 3-(3-morpholin-4-ylpropoxy)phthalonitrile (3) were found at m/z 272 [M+H]^+^. The absence of the C≡N stretches at 2230 (for 2) and 2228 cm^-1^ (for 3) in the IR spectra of the Co(II)Pc and Mn(III)Pcs bearing (3-morpholin-4-ylpropoxy) groups confirms that the cyclotetramerization consisted. The IR spectra of the Co(II)Pc and Mn(III)Pcs bearing (3-morpholin-4-ylpropoxy) groups are very similar. ^1^H-NMR and ^13^C-NMR evaluations of the Co(II)Pc and Mn(III)Pcs were inhibited because of their paramagnetic characteristic [24]. MALDI-TOF mass spectra of Co(II)Pc and Mn(III)Pcs (2a, 2b, 3a, 3b) confirmed the structures, with the molecular ion being easily identified at 1144.09 [M]^+^, 1140.42 [M-Cl]^+^ (Figure 2), 1144.12 [M]^+^ and 1140.63 [M-Cl]^+^, respectively. The UV-Vis spectra of Co(II)Pc and Mn(III)Pcs (2a, 2b, 3a, 3b) in CHCl_3_ are shown in Figure 3. UV-Vis spectra of Co(II)Pc and Mn(III)Pcs (2a, 2b, 3a, 3b) showed single Q band absorption of π → π∗ transitions at 673, 735, 694, 764 nm, respectively. B bands of Co(II)Pc and Mn(III)Pcs (2a, 2b, 3a, 3b) were appeared in the UV region at 324, 384, 309, (355, 332) nm, respectively. On the other hand, 2b and 3b have an absorption band at 530 nm for 2b and 542 nm for 3b, interpreted as a charge transfer absorbtion [25].

### 3.2. Electrochemical studies

**Figure 4:**
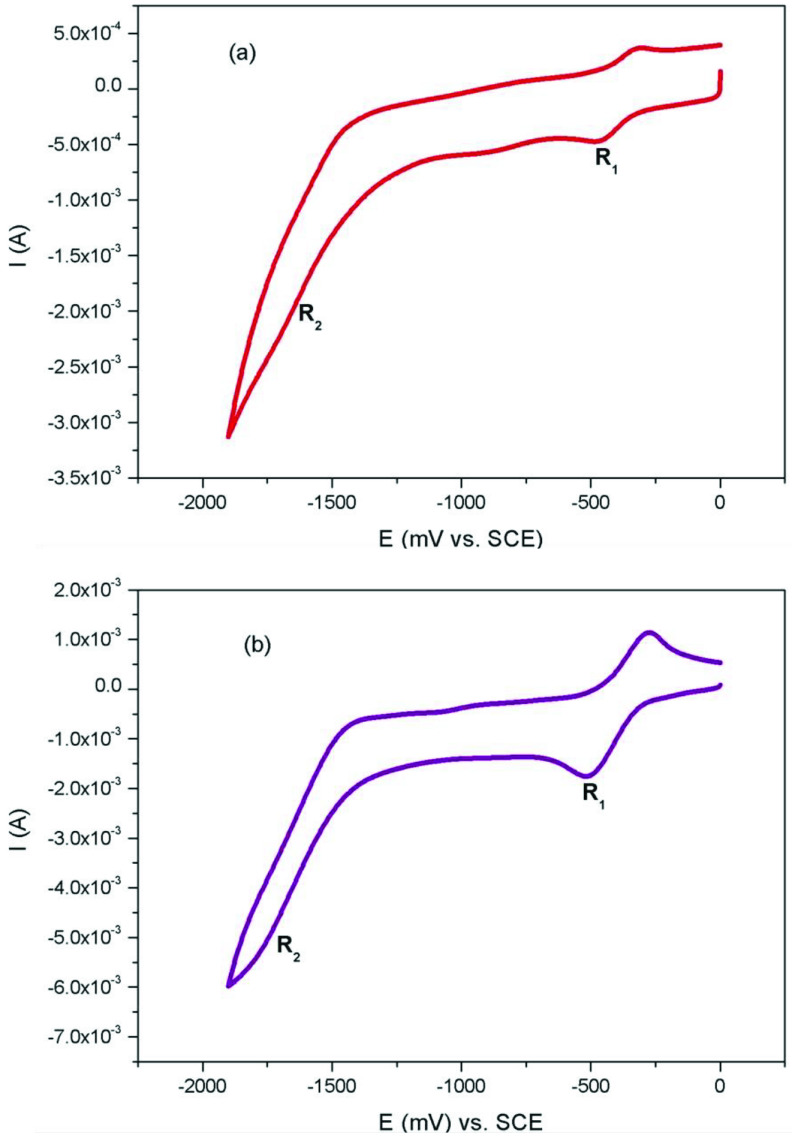
(a) CV graph of 2a. (b) CV graph of 3a.

**Figure 5:**
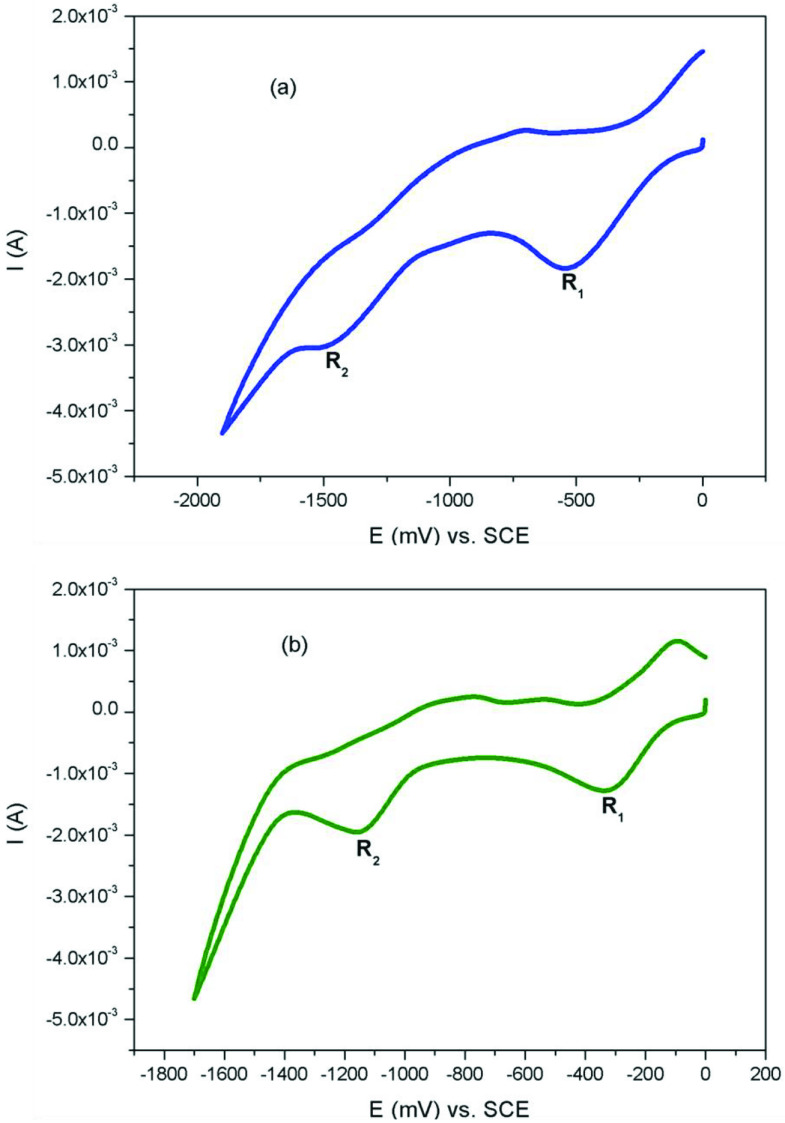
(a) CV graph of 2b. (b) CV graph of 3b.

The electrochemistry of Co(II)Pc and Mn(III)Pcs (2a, 2b, 3a, 3b) were obtained in DCM using a (DCM)/(TBAP) electrolyte system on a Pt working electrode. The electrochemical data were listed in Table. Figure 4a and Figure 4b show the CV responses of 2a, 3a in DCM/TBAP electrolyte system. 2a and 3a exhibited 2 reduction labelled as R_1_ (E_1/2_ = –0.39 V for 2a, E_1/2_ = –0.40 V for 3a) and R2 (E_1/2_ = –1.54 V for 2a, E_1/2_ = –1.57 V for 3a) in DCM/TBAP electrolyte system. Co^II^ can reduce before Pc ring, thus the R_1_ process of 2a and 3a at E_1/2_ = –0.39 V and –0.40 V is easily assigned to the Co^II^ /Co^I^ reduction reaction of the complexes [26]. Figure 5a and Figure 5b show the CV responses of 2b, 3b in DCM/TBAP electrolyte system. 2b and 3b exhibited 2 reduction labelled as R_1_ (E_1/2_ = –0.25 V for 2b, E_1/2_ = –0.21 V for 3b) and R2 (E_1/2_ = –1.29 V for 2b, E_1/2_ = –1.09 V for 3b) in DCM/TBAP electrolyte system. The first reduction can be assigned to [Cl-Mn^III^ Pc^-2^ ] / [Cl-Mn^II^ Pc^-2^ ]^-1^ because of the redox active manganese metal ion [27]. Then second reduction can be assigned to [Mn^II^ Pc^-2^ ] / [Mn^I^ Pc^-2^ ]^-1^ couple [28]. After first reduction, [Cl-Mn^II^ Pc^-2^ ]^-1^ species release axial chloride ion. Similar results were appeared for Co(II)Pc and Mn(III)Pcs in literature [29,30].

**Table:** Voltammetric data of the Pcs. All voltammetric data were given versus SCE

Pcs		Oxidations	Reductions	
2a	^a^E_1/2_	1.32^c^	–0.39	–1.54
	^b^ΔE_p_ (mV)	-	113	140
2b	^a^E_1/2_	1.38^c^	–0.25	–1.29
	^b^ΔE_p_ (mV)	-	175	166
3a	^a^E_1/2_	1.36^c^	–0.40	–1.57
	^b^ΔE_p_ (mV)	-	96	131
3b	^a^E_1/2_	1.33^c^	–0.21	–1.09
	^b^ΔE_p_ (mV)	-	122	151

^a^: E_1/2_ values ((E_pa_ + E_pc_)/2) were given versus SCE at 0.100 Vs^-1^ scan rate. ^b^: ΔE_b_ = E_pa_-E_pc_. ^c^:E_pa_ of first CV cycle.

**Figure 6:**
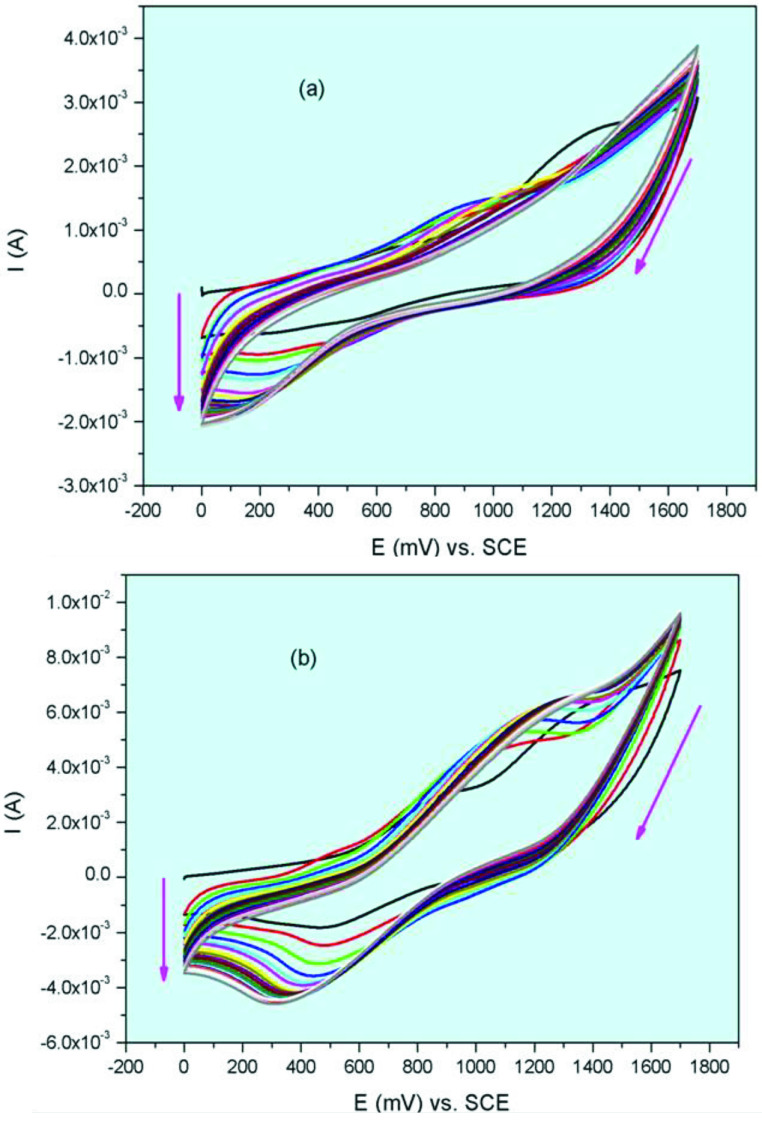
(a) Repetitive CVs of 2a. (b) Repetitive CVs of 3a.

**Figure 7:**
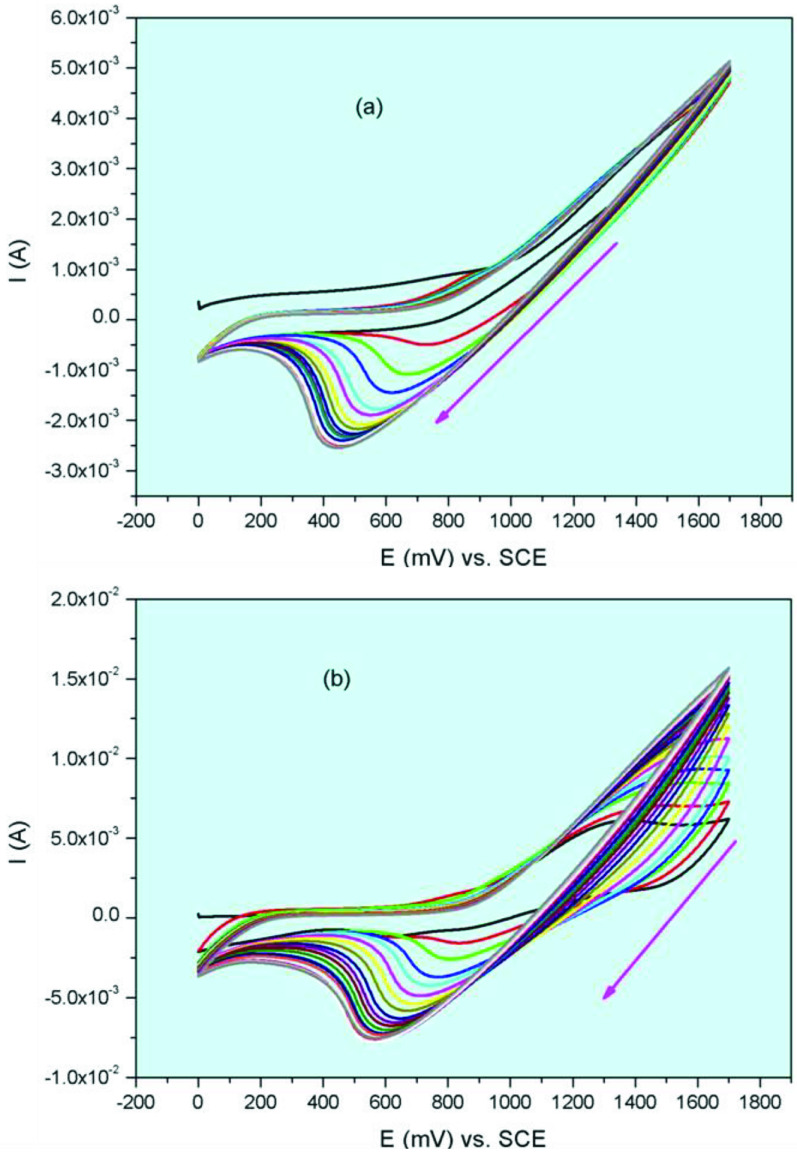
(a) Repetitive CVs of 2b. (b) Repetitive CVs of 3b.

While peripheral, nonperipheral tetra-(3-morpholin-4-ylpropoxy) group substituted Co(II)Pc and Mn(III) Pcs (2a, 2b, 3a, 3b) illustrate widespread reduction reactions during the cathodic scans, Co(II)Pc and Mn(III)Pcs were electropolymerized on the working electrode during the anodic scans. Figure 6 shows the CV responses of peripheral, nonperipheral tetra-(3-morpholin-4-ylpropoxy) group substituted cobalt(II) phthalocyanines (2a and 3a) during repetitive CV cycles. When Figure 6a is examined, the onset oxidation potential of peripheral tetra-(3-morpholin-4-ylpropoxy) group substituted cobalt(II) phthalocyanine 2a at around 1.32 V is observed, whereas the onset potential of the nonperipheral tetra-(3-morpholin-4-ylpropoxy) group substituted cobalt(II) phthalocyanine 3a has been determined at around 1.36 V (Figure 6b). In the following cycles, the oxidation peak currents raised and there was a small shift in the oxidation peaks. This suggests that the Co(II)Pc and Mn(III)Pcs incur polymerization in each scan and deposit onto the electrode surface. Figure 7 shows the CV responses of peripheral, nonperipheral tetra-(3-morpholin-4-ylpropoxy) group substituted manganese(III) chloride phthalocyanines (2b and 3b) during repetitive CV cycles. When Figure 7a is examined, the onset oxidation potential of peripheral tetra-(3-morpholin-4-ylpropoxy) group substituted manganese(III) chloride phthalocyanine 2b at around 1.38 V is observed, whereas the onset potential of the nonperipheral tetra-(3-morpholin-4-ylpropoxy) group substituted manganese(III) chloride phthalocyanine 3b has been determined at around 1.33 V (Figure 7b). Similar to 2a and 3a, peripheral, nonperipheral tetra-(3-morpholin-4-ylpropoxy) group substituted manganese(III) chloride phthalocyanines (2b and 3b) show the electropolymerization process. Morpholine derivatives generally polymerize during the oxidation reaction. For example, cobalt, titanium, manganese phthalocyanines bearing [(5-{[(1E)-(4-morpholin-4-ylphenyl)methylene]amino}-1-naphthyl)oxy] substituent was electropolymerized on GCE [20]. When compared with [(5-{[(1E)-(4-morpholin-4-ylphenyl)methylene]amino}-1-naphthyl)oxy] cobalt, titanium, manganese phthalocyanines, redox processes of the studied phthalocyanines in present work, are generally compatible with a small potential difference. Because of the electropolymerization properties, peripheral, nonperipheral tetra-(3-morpholin-4-ylpropoxy) group substituted cobalt(II), manganese(III), phthalocyanines (2a, 2b, 3a, 3b) may be a good nominee in electrochemical fields such as electrocatalysts, electrochromic applications.

## Conclusion

As a conclusion, synthesis and electrochemistry of Co(II)Pc and Mn(III)Pcs containing (3-morpholin-4-ylpropoxy) groups at peripheral and nonperipheral positions have been presented in this study. Cyclic voltammetry was used in order to determine electrochemistry of Co(II)Pc and Mn(III)Pcs containing (3-morpholin-4-ylpropoxy) groups at peripheral and nonperipheral positions. According to the electrochemical results, Co(II)Pc and Mn(III)Pcs gave common reduction reactions. On the other hand, Co(II)Pc and Mn(III)Pcs containing (3-morpholin-4-ylpropoxy) groups at peripheral and nonperipheral positions were plated on Pt electrodes with the oxidation for polymerizable morpholino groups. Electropolymerization renders phthalocyanine, a valuable material for the production of different electrochemical applications, for example electrocatalytic, electrochromic, and electrosensing applications.

## References

[1] (2012). Novel water-soluble metallophthalocyanines supported on cotton fabric. Coloration Technology.

[2] (2017). Zinc(II) phthalocyanine fused in peripheral positions octa-substituted with alkyl linked carbazole: Synthesis, electropolymerization and its electro-optic and biosensor applications. Biosensors Bioelectronics.

[3] (1998). Metallophthalocyanines: Gas sensors, resistors and field effect transistors. Coordination Chemistry Reviews.

[4] (2019). Synthesis of peripherally tetra substituted neutral azophenoxy zinc phthalocyanine and its application in bulk hetero junction solar cells. Journal of Molecular Structure.

[5] (2018). -carboxylic acid as a new anchoring group for phthalocyanine-sensitized solar cells. Solar Energy.

[6] (2019). Palladium(II) octaalkoxy- and octaphenoxyphthalocyanines: Synthesis and evaluation as catalysts in the Sonogashira reaction. Journal of Catalysis.

[7] (2019). Comparative nonlinear optics and optical limiting properties of metallophthalocyanines. Inorganica Chimica Acta.

[8] (2018). Liquid-crystalline phthalocyanine with short intercolumnar distance and variation of the liquid crystallinity induced by square-planar metal ions. Chemistry Letters.

[9] (2017). -tetrafluorophenoxy)ethoxy\textbraceright substituted cobalt(II), iron(II) metallophthalocyanines: Synthesis and their electrochemical, catalytic activity studies. textbraceleft 2-(2.

[10] (2013). Synthesis, characterization and investigation of homogeneous oxidation activities of peripherally tetra-substituted Co(II) and Fe(II) phthalocyanines: Oxidation of cyclohexene. Journal of Molecular Catalysis A Chemical.

[11] (2019). Investigation of novel substituted zinc and aluminium phthalocyanines for photodynamic therapy of epithelial breast cancer. Dyes and Pigments.

[12] (2017). Synthesis and investigation of photophysicochemical properties of novel ketone-substituted gallium (III) and indium (III) phthalocyanines with high singlet oxygen yield for photodynamic therapy. Journal of Luminescence.

[13] (2016). Electrocatalytic activity of nanocomposites of sulphur doped graphene oxide and nanosized cobalt phthalocyanines. Electroanalysis.

[14] (2014). Electrode modification based on "click electrochemistry" between terminal-alkynyl substituted cobalt phthalocyanine and 4-azidoaniline. Sensors and Actuators B Chemical.

[15] (2019). Electropolymerized octabenzimidazole phthalocyanine as an amperometric sensor for hydrazine. Journal of Electroanalytical Chemistry.

[16] (2016). Substituent effects to the electrochromic behaviors of electropolymerized metallophthalocyanine thin films. Journal of Solid State Electrochemistry.

[17] (2018). Spin-active, electrochromic, solvent-free molecular liquid based on double-decker lutetium phthalocyanine bearing long branched alkyl chains. Chemistry: An Asian Journal.

[18] (2002). Electrocatalytic activity of cobalt phthalocyanine stabilized by different matrixes. Analytical and Bioanalytical Chemistry.

[19] (2014). Synthesis, electrochemistry, spectroelectrochemistry and electropolymerization of metal-free and metallophthalocyanines. Polyhedron.

[20] (2017). Electropolymerization of metallophthalocyanines carrying redox active metal centers and their electrochemical pesticide sensing application. Electroanalysis.

[21] (2008). The synthesis and electrochemical behaviour of water soluble manganese phthalocyanines: Anion radical versus Mn(I) species. Inorganic Chemistry Communications.

[22] (2017). Non-peripherally tetrasubstituted phthalocyanines: Synthesis, characterization and, photophysical investigation. Journal of Organometallic Chemistry.

[23] (2016). Mono- and tetra-substituted zinc(II) phthalocyanines containingmorpholinyl moieties: Synthesis, antifungal photodynamic activities,and structure-activity relationships. European Journal of Medicinal Chemistry.

[24] (2019). Synthesis and electrochemical properties of peripheral, non-peripheral tetra [2-(3,5-diphenyl-. 1H-1.

[25] (2010). The effects of point of substitution on the electrochemical behavior of new manganese phthalocyanines, tetra-substituted with diethylaminoethanethiol. Inorganica Chimica Acta.

[26] (2015). An effect of the substituent position and metal type on the electropolymerization properties of chalcone substituted metallophthalocyanines. Dalton Transactions.

[27] (2017). -yl)phenoxy) substituted cobalt, iron and manganese phthalocyanines: Synthesis and electrochemical analysis. Tetra(3-(1.

[28] (2015). Electrochemical, electrocatalytic dioxygen reducing and dielectric relaxation properties of non-peripheral tetra-2,3-dihydro-1H-inden-5-yloxy substituted phthalocyanines. Journal of the Electrochemical Society.

[29] (2017). Synthesis, characterization and investigation of electrochemical and spectroelectrochemical properties of peripherally and non-peripherally tetra 2-methyl-5-benzothiazole substituted nickel(II), copper(II) and cobalt(II) phthalocyanines. Synthetic Metals.

[30] (2015). -Triazole-substituted metallophthalocyanines carrying redox active cobalt(II), manganese(III), titanium(IV) center and their electrochemical studies. Synthetic Metals.

